# Description of Bernardella vulneris gen. nov., sp. nov., assignable to the family Aerococcaceae, isolated from human clinical samples

**DOI:** 10.1099/ijsem.0.007180

**Published:** 2026-08-11

**Authors:** Anne-Marie Bernier, Dmytro Lyubashenko, Ana Luisa Pacheco, Mark Unger, Marc-Christian Domingo, Jennifer Tanner

**Affiliations:** 1 National Microbiology Laboratory, Public Health Agency of Canada, Winnipeg, MB, Canada; 2 Department of Biology, Université de Saint-Boniface, Winnipeg, MB, Canada; 3 Laboratoire de Santé Publique du Québec, Institut national de santé publique du Québec, Ste-Anne-de-Bellevue, QC, Canada

**Keywords:** *Bernardella vulneris*, human clinical isolates, wound infections

## Abstract

Eleven novel Gram-stain-positive, catalase- and oxidase-negative coccal bacterial isolates recovered mostly from the wounds of patients located in three Canadian provinces were extensively studied. Phenotypic analysis and phylogenetic analysis based on both the 16S rRNA gene and whole-genome sequence place them in the family *Aerococcaceae* and differentiate them from genera in the closest families *Enterococcaceae* and *Carnobacteriaceae* in the order *Lactobacillales*. The G+C content of the isolates was on average 42.15 mol% with genome sizes ranging from 1.87 to 2.04 Mb. The average nucleotide identity and average amino acid identity values to other members of the family *Aerococcaceae* were below 69 and 62%, respectively, placing the isolates into a new genera. Phosphatidylserine is predicted to be synthesized based on the presence of the gene phosphatidylserine synthase in the annotated genome (E.C. 2.7.8.8). *In silico* analysis predicts the presence of l-lysine as the main diamino acid in the peptidoglycans. Major cellular fatty acids included C_16:0_, C_18:0_ and C_18:1_ *ω*9c. These 11 clinical strains were ultimately deemed to represent a novel genus and species, for which the name *Bernardella* gen. nov. and *Bernardella vulneris* sp. nov. is proposed and the type strain is NML130460^T^ (=LMG 32739^T^=NCTC 14840^T^).

## Introduction

The family *Aerococcaceae* currently consists of 12 genera and 32 species with validly published names and 1 genus whose name is not yet validly published (https://lpsn.dsmz.de/family/aerococcaceae accessed 3 July 2025) [1]. The genera *Ruoffia*, *Hutsoniella* and *Falseniella* were the most recent additions to the family in 2021 [2]. The type genus, *Aerococcus*, consists of 13 species, with *Aerococcus kribbianus* being the most recently described in 2024 [3] and the type species being *Aerococcus viridans*. All species with validly published names except *Aerococcus vaginalis* and *Facklamia fermenti* have one or more genomes deposited in the NCBI database; these range from a G+C content of 35.5–47 mol% and genome size of 1.36 Mb (*Aerococcus suis*) to 3.2 Mb (*Fundicoccus culcitae*) (https://www.ncbi.nlm.nih.gov/genome, accessed 3 July 2025). The genera exhibit a wide variety of characteristics, but briefly, the members of this family are considered non-motile, non-sporulating Gram-stain-positive ovoid cocci or coccobacilli. They are facultatively anaerobic, oxidase-negative and generally catalase-negative with the exception of *Suicoccus acidiformans* [4, 5]. Chemotaxonomic properties for many genera in this family include branched-chain saturated cellular fatty acids (CFAs) and l-lysine diamino acid peptidoglycan types.

The Special Bacteriology Unit at the National Microbiology Laboratory (NML) in Winnipeg, Canada, is a reference laboratory that routinely receives clinically derived bacterial samples from provincial public health labs for identification. These were typically unidentifiable by referring laboratories using standard methods, such as matrix-assisted laser desorption/ionization time-of-flight (MALDI-TOF) MS. Eleven such unidentifiable isolates were referred for identification, and subsequent phylogenetic and phenotypic analyses showed them to be a novel genus forming a distinct monophyletic clade in the family *Aerococcaceae*. The names *Bernardella* gen. nov. and *Bernardella vulneris* sp. nov. are proposed and NML13460^T^ is the type strain.

## Isolates and sample descriptions

In this study, 11 clinically derived strains (Table 1) recovered between 2013 and 2021 from patients in three Canadian provinces were found to be unidentifiable with respect to existing taxa using MALDI-TOF MS and 16S rRNA gene sequencing. Nine were from wounds or biopsies, and two strains were from unknown sources. No additional clinical information was available. The studied bacteria were cultured on Columbia blood agar base (CBAB) with 5% sheep blood at 35 °C with 5% CO_2_ for 24 h and were Gram-stain-positive cocci, found mainly in pairs.

**Table 1.** Sources of strains studied and NCBI accession numbers

				NCBI accession no.		
Isolate	Source	Year isolated	Province	16S	WGS	Assembly
NML130460^T^	Deep wrist wound	2013	NB	ON756181	JAMXJD01	GCA 026131645.1
NML160702	Knee pus	2016	QC	ON756180	JAMXJE01	GCA 026131615.1
NML171108	Foot wound	2017	BC	ON756179	JAMXJF01	GCA 026131585.1
NML180378	Index finger pus	2018	QC	ON756178	JAMXJG01	GCA 026131595.1
NML190073	Cutaneous biopsy	2019	QC	ON756177	JAMXJH01	GCA 026131555.1
NML190938	Unknown	2019	QC	ON756176	JAMXJI01	GCA 026131545.1
NML191219	Thumb bone/joint	2019	BC	ON756175	JAMXJJ01	GCA 026131505.1
NML191292	Hand abscess pus	2019	QC	ON756174	JAMXJK01	GCA 026131525.1
NML201209	Lower limb wound	2020	QC	ON756173	JAMXJL01	GCA 026131485.1
NML201296	Hand pus	2020	QC	ON756172	JAMXJM01	GCA 026131445.1
NML210727	Unknown	2021	QC	ON756171	JAMXJN01	GCA 026131455.1

WGS, Whole genome sequence.

## 16S rRNA gene sequencing and analysis

The phylogenetic position of the isolates was initially determined using 16S rRNA gene sequencing as previously described [6]. The sequencing generated a near full-length gene sequence (1,450–1,500 bps) which was then used in the NCBI blastn analyses for identification (ID). blastn match IDs of 100% were obtained to *Globicatella* sp. feline oral taxon 122 strain 7138 (accession KM461962.1) and to *Lactobacillales* bacterium 1247 (HQ115584.1), while *Globicatella* sp. feline oral taxon 122 Clone TE083 (KM462016.1) had a 99.8% ID. The blastn results to the refseq RNA database and to the reference genome database revealed no close matches, with *Globicatella sanguinis* (NR_104716.1) being the closest at only 93.34% identity.

The 16S rRNA gene sequences from the 11 NML strains had ≥99.45% identity to each other by blastn (Table S1A, B, available in the online Supplementary Material), with strain NML130460 sharing ≥99.66% identity with the other NML strains (Table S1A, B). Strain NML130460^T^, the first strain isolated in this group, was designated the type strain of this new genus and used as the comparator in some downstream analyses.

The 16S rRNA gene sequences from the type species of the 13 genera in the family *Aerococcaceae* were retrieved directly from the LPSN (https://lpsn.dsmz.de), and blastn was applied to calculate the percent identity to strain NML130460^T^ (Table 2). These had ≤93.3% identity to NML130460^T^, indicating that the cluster of NML strains form a novel taxonomic group. The 16S rRNA gene sequences of the type strains of the 32 species in the *Aerococcaceae* obtained from the LPSN were also compared by blastn, and all strains had ≤93.3% ID to NML130460^T^ (Table S1C).

**Table 2.** Overall genome relatedness indices and 16S blastn results for *Aerococcaceae* type species compared to NML130460^T^. ANIb was calculated through the JSpeciesWS using the built-in database for reference strains [16]. The dDDH was calculated from the Genome-to-genome database [1], and AAI was estimated using the AAI calculator from Kostas lab [19]. 16S blastn results are % identity

	16S blast	ANIb	dDDH	AAI
*Abiotrophia defectiva* DSM 9849^T^	88.683	68.53	27.3	62.13
*A. viridans* CCUG 4311^T^	92.812	66.67	22	48.91
*Dolosicoccus paucivorans* DSM 15742^T^	91.707	66.95	22.6	55.81
*Eremococcus coleocola* DSM 15696 ^T^	90.469	66.42	23.8	56.45
*Facklamia hominis* CCUG 36813^T^	91.396	66.25	24	53.77
*Falseniella ignava* CCUG 37419^T^	91.844	66.72	20.7	54.19
*Fundicoccus ignavus* DSM 109652^T^	92.74	68.46	21.1	59.85
*G. sanguinis* NBRC 15551 ^T^	93.338	68.72	23	59.38
*Hutsoniella sourekii* ATCC 700629^T^	91.382	65.98	19.7	55.10
*Ignavigranum ruoffiae* DSM 15695^T^	91.195	66.48	23.4	55.01
*Ruoffia tabacinasalis* CCUG 30090^T^	91.307	67.3	23.2	56.89
*S. acidiformans* ZY16052^T^	91.352	66.58	26.1	55.41
*‘Vaginisenegalia massiliensis’* Marseille-P5643^T^	92.779	68.51	27.4	60.40

AAI, Average Amino Acid Identity; ANIb, Average nucleotide identity by BLAST; dDDH, digital DNA-DNA hybridization.

**Fig. 1.**
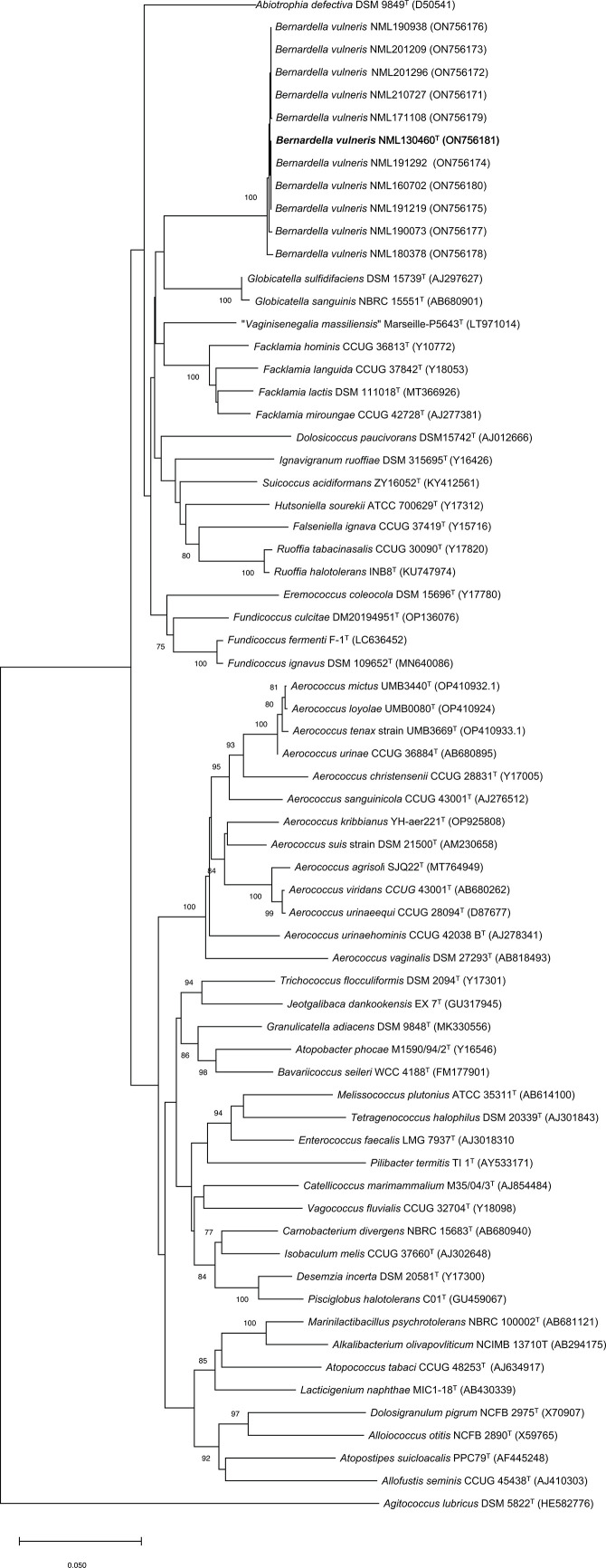
Evolutionary analysis of the 16S rRNA gene from the clinical strains and species from the *Aerococcaceae, Carnobacteriaceae* and *Lactococcaceae* inferred using the maximum-likelihood method and Tamura–Nei model [10]. The tree with the highest log likelihood (−20,288.51) is shown to scale, with branch lengths measured in the number of substitutions per site. There were a total of 1,575 positions in the final dataset. Evolutionary analyses were conducted in mega X with alignments performed using Clustal W [7]. Tree scale bar represents substitution per nucleotide position.

To ascertain the phylogenetic position of the NML strains, the 16S rRNA gene sequences from the 32 species in the *Aerococcaceae*, the 11 NML strains and the type species from genera in the more closely related families *Carnobacteriaceae* and *Enterococcaceae* from the order *Lactobacillales* were aligned in mega X [7] using Clustal W [8] and the maximum-likelihood algorithm and Tamura–Nei model [9, 10]. The results placed them in the *Aerococcaceae* family (Fig. 1). Both the neighbour joining and minimum evolution phylogenetic trees inferred from the same mega X alignment of the 16S rRNA genes (Figs S1 and S2) show similar tree topologies with the NML strains clustering together within the *Aerococcaceae* on a separate branch.

**Fig. 2.**
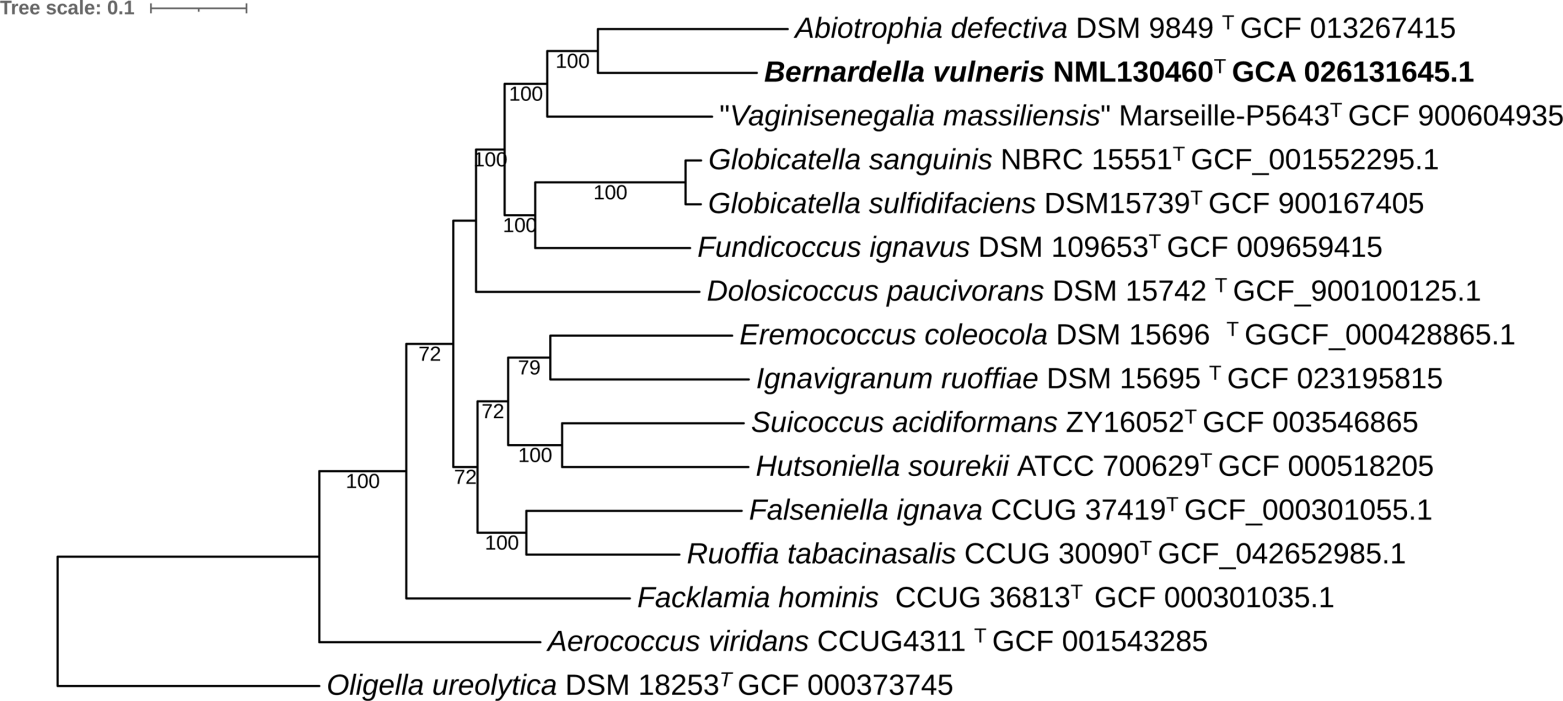
Molecular phylogenomic analysis of isolates using default parameters (100 core genes) by autoMLST [21] for *B. vulneris* NML130460^T^ and type species of the *Aerococcaceae* genera. Bootstrap values were calculated for 100 subsets at branch nodes. *O. ureolytica* DSM 18253^T^ was the outgroup. Tree scale bar 0.1 substitutions per nucleotide position.

## Whole-genome sequencing and analysis

All NML strains were analysed using whole-genome sequencing (WGS). DNA extraction and paired-end (PE) libraries were prepared as previously described [11]. Read quality was assessed using FastQC (galaxy v0.72 https://www.bioinformatics.babraham.ac.uk/projects/fastqc/), and assembly of untrimmed PE reads was performed with the Shovill pipeline using SPAdes (v3.13.1) as the assembler [12]. Assembly quality was assessed using Quast (Galaxy v5.0.2) [13], and draft genomes were annotated using PGAP (v6.1) (Table S2) [14]. The genomes ranged in size from 1.858 to 2.069 Mb with an average G+C content of 42.15 mol%. All genomes were found to have CRISPR arrays with ≥4 spacers each (Table S3) using the CRISPRCasFinder program (https://crisprcas.i2bc.paris-saclay.fr/CrisprCasFinder/Index) [15]. The number of spacers and the highly diverse sequences suggest that the isolates have been infected with numerous different bacteriophages, with one CRISPR array in isolate NML160702 having a very large array with 192 spacers. Eight different consensus repeat sequences were identified in the eleven isolates, and neither the spacer sequences nor the repeat sequences were found in the spacer database and so appear to be novel.

**Table 3.** CFA composition of NML130460^T^ and closely related genera [4, 5]

CFA in order of elution equivalent chain length (ECL)	1	2	3	4	5	6	7	8	9
C_10:0_	0.6	0.5	NR	NR	NR	NR	NR	NR	NR
C_12:0_	2.06	1	0.4	1.2	0.5	0.5	0.5	0.5	1.3
C_14:0_	4.33	8.6	4.1	9	6.1	4.0	6.3	7.0	**10.6**
C_15:0_ anteiso	–	–	0.3	–	0.4	0.5	4.2	1.0	0.2
C_16:0_	**22.9**	**30.2**	**25.4**	**33.4**	**52.9**	**44.1**	**52.0**	**56.8**	**38.1**
C_17:0_	1.4	0.5	NR	NR	NR	NR	NR	NR	NR
C_18:0_	**14**	**11.9**	**13.8**	4.8	5.0	5.8	3.9	3.1	2.4
C_19:1_ iso1	–	–	–	1.1	–	1.8	–	–	0.2
C_16:1_ *ω*9c	2.7	4.3	**15.3**	**18.1**	–	7.9	–	–	**18.4**
C_17:1_ iso *ω*5c	–	0.7	0.3	2.2	0.9	3.4	0.6	0.8	0.5
C_18:1_ *ω*9c	**22.4**	**17**	9.3	**12.1**	**11.8**	**10.1**	9.3	**10**	**12.7**
C_20:1_ *ω*9c	–	–	7.2	–	–	–	–	–	–
C_20:2_ *ω*6,9c	0.86	–	NR	NR	NR	NR	NR	NR	NR
C_20:4_ *ω*6,9,12,15c	1.11	0.6	NR	NR	NR	NR	NR	NR	NR
C_13:0_ 2-OH	–	–	–	–	–	0.6	–	–	–
C_15:0_ 2-OH	–	–	–	–	–	0.9	–	–	–
Summed features*									
3	6.6	4.5	1.4	5.6	2.9	3.1	2.5	2.8	2.1
5	**12.7**	**16.4**	1.6	5.6	**15.9**	9.4	**13.2**	**13.3**	9.6
7	–	–	**16.3**	–	–	–	–	–	–
8	5.3	2.9	1.7	3.8	1.2	1.9	1.2	1.2	0.8
9	0.4	0.9	0.5	1.7	–	–	–	–	1

1, NML130460T; 2, *G. sanguinis* NBRC 15551T; 3,* A. viridans* CCUG 4311T; 4, *Abiotrophia defectiva* DSM 9849T; 5, *Facklamia hominis* CCUG 36813T; 6, *Dolosicoccus paucivorans* DSM 15742T; 7, *Eremococcus coleocola* DSM 15696T; 8, *Ignavigranum ruoffiae* DSM 15695T; 9, *S. acidiformans* ZY16052T.

*Summed feature 3, C_16:1_ *ω*7с/C_16:1_ *ω*6с; summed feature 5 C_18:2_* ω*6,9с/C_18:0_ ante; summed feature 7 unknown 18.85 /C_19:1_ *ω*6с; summed feature 8 C_18:1_ *ω*7с/C_18:1_ *ω*6с; summed feature 9 C_17:1_ iso *ω*9с/C_16:0_ 10-methyl.

Values in bold are significant amounts, >10%.

NR, Not reported.

Pairwise average nucleotide identity by blast (ANIb) values were calculated through JSpeciesWS (https://jspecies.ribohost.com/jspeciesws) using the type strain genome sequences from genomes in the pipeline’s reference database, GenomeDB, to assess overall genome relatedness [16]. Whole-genome sequences for *A. vaginalis* and *Fundicoccus fermenti* were not publicly available and so could not be included in WGS analyses. These species were however shown not to be closely related to the study isolates by 16S analysis (Table S1C, Fig. 1). ANIb values of 95–96% were used to delineate species’ boundaries [17]. The ANIb values of 10 NML strains to NML130460^T^ were between 97 and 98.16% (Table S1B), and the values between the 30 species of the *Aerococcaceae* family and NML130460^T^ were 64.34–68.9% (Tables 2 and S1C), supporting the proposal that the NML strains form a new separate clade within the family. The digital DNA–DNA hybridization (dDDH) values were also assessed using the Genome-to-Genome Distance Calculator (GGDC) v3.0 (https://ggdc.dsmz.de) [1]. The study strains had distance values <30% to all species in the *Aerococcaceae* family using GGDC formula 2, which is recommended for draft genomes (Tables 2 and S1C). The values of the ten other study strains to NML130460^T^ were ≥75.3% with formula 2 (Table S1B). Genomes are considered to be of the same species when the dDDH values (formula 2) are >70% [18]; therefore, these data confirm the clustering of this group of isolates as a novel taxon.

The average amino acid identity tool (AAI-matrix tool) from Kostas lab [19] (http://enve-omics.ce.gatech.edu/g-matrix/) was used to calculate average amino acid identities (AAIs) between NML130460^T^ and the other NML strain as well as to all *Aerococcaceae* strains from FASTA amino acid files produced using Prokka annotation [20]. The cutoff proposed for species-level differentiation is ≥90% for AAI (http://enve-omics.ce.gatech.edu/g-matrix/faq), and 74–76% is proposed as the cutoff for genus differentiation [21]. The comparative AAI values of the ten NML strains to NML130460^T^ were ≥96% (Table S1B), and NML130460^T^ had ≤62.1% AAI values to other genera of the *Aerococcaceae* family (Table S1C) [19].

The overall genome relatedness indices (ANIb, AAI and dDDH) and 16S rRNA gene blastn identity of all species of *Aerococcaceae* to NML130460^T^, as well as the values obtained for each NML strain to NML130460^T^, confirm that the clinical strains in this study form a monophyletic cluster and represent a novel genus.

As a further confirmation of the position of the isolates within the *Aerococcaceae* family, an autoMLST analysis [22] was performed with the IQ-TREE ultrafast bootstrap analysis (1,000 replicates) using the NML130460^T^ genome and the *Aerococcaceae* type species as the query genomes. The outgroup, as proposed by the algorithm, was *Oligella ureolytica* DSM 18253^T^. The 100 default multilocus sequence typing genes selected by the online workflow script were used in the analysis. The maximum-likelihood tree inferred from the multi-locus sequence analysis (Fig. 2) again confirms the findings from the 16S analysis, which positions the NML strains in the *Aerococcaceae* family on a distinct branch.

**Fig. 3.**
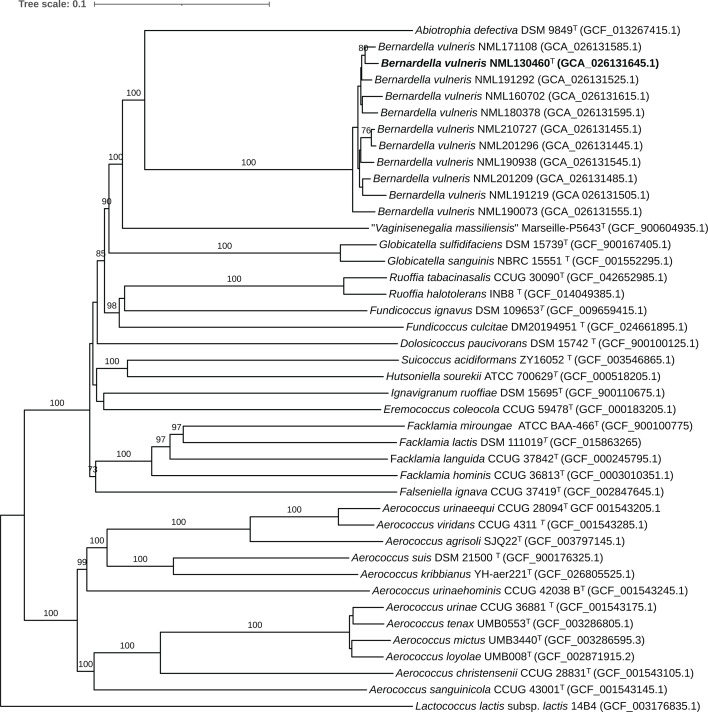
Tree inferred through TYGS [1] with FastME 2.1.6.1 [22] from whole-proteome-based GBDP distances. The branch lengths are scaled via GBDP distance formula d5. Branch values are GBDP pseudo-bootstrap support values >60% from 100 replications, with an average branch support of 72.6%. The tree was midpoint-rooted.

The draft genomes of all NML strains were submitted to the TYGS pipeline [1] (https://tygs.dsmz.de/) along with those from the *Aerococcaceae* family, and the GBDP WGS ML tree was generated from these genomes. The branch support for this tree was found to be very low (26%) (data not shown) because of the large diversity in the family, and so the tree inferred with FastME [23] from the whole proteome-based GBDP distance was produced. This tree (Fig. 3) had a branching support of greater than 72.6%, and the NML strains cluster together on a separate branch of the tree from the current genera of the family. The outgroup for this analysis was the reference genome of *Lactococcus lactis* subsp*. lactis* strain 14B4^T^, and the tree is rooted to the mid-point.

The genomes were analysed using the Resistance Gene Identifier of the Comprehensive Antibiotic Resistance database through the web portal (RGI 6.0.3, CARD 4.0.0) [24]. There were no perfect hits to genes in the database; however, one strict match (% ID 99.52 with 92.48% coverage) to QnrB10 which confers fluoroquinolone resistance mainly in Gram-stain-negative bacteria was identified only in strain NML130460^T^. Antimicrobial susceptibility testing was performed on NML130460^T^ bacteria culture grown at 35 °C on cation-adjusted Mueller–Hinton broth supplemented with 2–5% lysed horse blood, for 24 h with 5% CO_2_, using the broth microdilution method with lyophilized Sensititre GPN3F and STP6F plates (Thermo Fisher) [25]. Only MICs are reported (Table S4) as there are no interpretive criteria for *Aerococcaceae* other than *Abiotrophia* spp. and *Aerococcus* spp. (Tables 1 and 2 of CLSI M45 third edition [25]); however, if these criteria were applied, the isolate was susceptible to all antimicrobials tested.

**Table 4.** Biochemical test results from NML130460^T^ and those reported from closely related strains [4, 5] obtained via API ZYM, API Strep 20 and API 50 CH kits

	*Aerococcaceae* strains*													
Substrate/enzyme/reaction	1	2	3	4	5	6	7	8	9	10	11	12	13	14
Acetoin production (Voges–Proskauer)	−	nr	−	−	−	nr	nr	−	−	nr	−	nr	nr	nr
Hippuric acid hydrolysis	−	−	+(w)	−	+	+	+	+	+	+	−	−	+	nr
Pyrrolidonyl arylamidase	+	+	nr	nr	nr	nr	nr	nr	v	nr	+	nr	nr	nr
Leucine aminopeptidase	+	+	−	−		+	+	nr	−	−	+	−	nr	+
Arginine dihydrolase	+	−	−	−	+	+	−	+	−	−	+	−	−	nr
d-Glucose	+	+	+	+(w)	+	−	+(w)	−	+	+	+(w)	nr	nr	+
d-Maltose	+	nr	−	nr	nr	−	v	nr	nr	+	nr	−		−
d-Melibiose	−	−	−	−	−	−	−	−	+	−	−	−	+	nr
d-Saccharose (sucrose)	−	+	+	−	−	−	−	−	+	+	−	+	+	−
Aesculin ferric citrate	+	nr	+	−	−	−	−	−	+	−	−	−	nr	nr
d-Ribose	−	−	+(w)	−	−	−	−	−	+	−	−	nr	−	nr
d-Mannitol	−	nr	+	+(w)	−	−	−	−	+	+	nr	−	+	−
d-Sorbitol	−	−	−	+	−	−	−	nr	−	+	−	−	−	−
d-Lactose (bovine origin)	+/+	−	+	+(w)	−	−	−	−	+	−	−	nr	−	−
d-Trehalose	+/−	+	−	−	−	−	−	−	+	+	−	−	+	+
d-Raffinose	−/−	+	−	−	−	−	−	−	+	−	−	nr	+	−
Amidon (starch)	+/−	+	nr	−	−	+	nr	−	+	nr	−	nr	nr	nr
Glycogen	+	+	−	−	−	−	−	−	−	−	−	nr	−	nr
Acid phosphatase	−	nr	nr	v	nr	nr	nr	nr	nr	−	−	−	+	+
*N*-Acetyl-*β*-glucosaminidase	−	−	−	−	+	−	−	−	−	−	−	−	nr	−
*α*-Mannosidase	−	−	−	−	−	nr	nr	+	nr	nr	−	nr	nr	−
*β*-Glucosidase	−	−	−	−	−	−	−	v	+	−	−	−	−	+
Alkaline phosphatase	−	−	−	v	−	−	−	−	−	+/−	−	−	+	−
*β*-Galactosidase	−	+	−	−	−	+	−	+	+	−	−	v	−	−
*β*-Glucuronidase	−	nr	−	−	−	−	−	−	−	−	−	−	nr	−
*α*-Galactosidase	−	+	−	−	−	+	−	+(w)	+	−	−	+	+	+
Catalase	−	−	−	−	−	−	−	−	−	−	−	−	+	−
Oxidase	−	−	−	−	−	−	nr	−	−	nr	+	nr	−	−

+/−, Opposite reactions with different assays; v, variable; +(w), weakly positive.

*1, *B. vulneris* 130460T; 2,* Abiotrophia defectiva* DSM 9849T; 3,* A. viridans* CCUG 4311T; 4, *Dolosicoccus paucivoran*s DSM 15742T; 5,* Eremococcus coleocola* DSM 15742T; 6,* Facklamia hominis* CCUG 36813T; 7,* Falseniella ignava CCUG 37419T*; 8,* Fundicoccus ignavus* DSM 109651T; 9, *G. sanguinis* NBRC 15551T; 10,* Hutsoniella sourekii* ATCC 700629T; 11, *Ignavigranum ruoffiae* DSM 15695T; 12, *Ruoffia tabacinasalis *CCUG 30090T; 13, *S. acidiformans* ZY16052T; 14, ‘*Vaginisenagalis massiliensis*’ Marseille-P5643T.

## Chemotaxonomy

*In silico* analysis of the glycerophospholipid metabolism of NML strains as determined from KEGG (https://www.kegg.jp/blastkoala/) [26] identified the presence of genes encoding enzymes for the production of phosphatidyl-l-serine from CDP-diacyl glycerol (phosphatidylserine synthase; E.C. 2.7.8.8). The enzyme cardiolipin synthase (2.7.8.41) required for the production of cardiolipin (diphosphatidylglycerol) is also present; however, this pathway is missing the enzyme required for the production of the cardiolipin precursor in the NML isolates. The presence of other glycerophospholipids could not be established *in silico* (Fig. S3, results for NML130460^T^).

*In silico* analysis of diamino acid of peptidoglycan was performed as described by Fotedar *et al*. [27]. Most Gram-positive bacteria generally have l-lysine as the main diamino acid, and so it is predicted that the clinical isolates will concord with this [28]. However, some Gram-positive bacteria such as *Corynebacterium*, *Clostridium* and *Bacillus* can have diaminopimelic acid as the diagnostic diamino acid. The UDP-*N*-acetylmuramoyl-l-alanyl-d-glutamate-2,5-diaminopimelate ligase (EC: 6.3.2.7) (also referred to as MurE) protein sequence extracted from NML130460^T^ was aligned to that of strains belonging to the *Aerococcaceae* family and related organisms using mega X and the Clustal W algorithm. The maximum-likelihood phylogenetic tree derived from the alignment placed NML130460^T^ in the cluster of genera known to produce L-lysine such as *Globicatella*, *Abiotrophia*, *Facklamia* and *Ignavigramun* (Fig. S4). Strain NML 130460^T^ is therefore predicted to contain type A peptidoglycan, with l-lysine at the third position within the pentapeptide.

CFAs from NML130460^T^ were extracted and assayed as described previously [29]. Analysis was performed using the Sherlock Microbial Identification System (MIDI) v4.5. The CFA composition of strain NML130460T and those reported for the type species of the family *Aerococcaceae* are presented in Table 3, arranged in order of increasing chain length [4, 5].

The CFA profile of NML130460^T^ was composed predominantly (>10%) of the linear fatty acids, C_16:0_, C_18:0_ and C_18:1_ *ω*9c. This profile is consistent with its assignment to the family *Aerococcaceae* (Table 3). Published data indicate that members of the genera within this family characteristically possess C_16:0_ and C_18:1_ *ω*9c as the major CFAs [4, 5]. Strain NML130460^T^ also contained C_18:0_ (14%) as a major CFA, a feature shared with *G. sanguinis* and *A. viridans. Aerococcus* is the type genus of the family *Aerococcaceae* [1]. In contrast, other genera within this family typically contain this fatty acid in moderate amounts (<10%) [4, 5].

Clinical isolates were analysed by MALDI-TOF MS (Bruker Microflex LT) using extraction methods as outlined by the manufacturer [30]. Analysis was performed with Biotyper 4.2 software and compared to Bruker Daltonics Biotyper database (v12438) with score results being interpreted as described by the manufacturer. Main spectral profiles (MSPs) were prepared for all study strains using methods outlined previously [31]. These had high scores of 2.2–2.5 towards each other but no matches (scores <1.3) to MSPs found in Biotyper .12438. This database is, however, missing some well-known species with standing nomenclature; therefore, while no matches were found, we cannot ascertain that these spectra could be sufficiently unique as to permit the identification of this taxon to the species level.

## Growth and physiology

Isolates formed round, entire, convex, grey, 0.5 mm, non-haemolytic colonies on CBAB with 5% sheep blood after 24-h incubation at 35 °C with 5% CO_2_. They were facultative anaerobes, growing to 2+/3+ in 24 h at 37 °C AnO_2_, 37 °C 5% CO_2_, 25 °C O_2_ and 42 °C O_2_ on 5% Columbia base sheep blood agar. Ideal growth conditions were 35 °C with 5% CO_2_. The isolates tolerated salinity with scant growth on nutrient broth (NB) with 6.5% (w/v) NaCl after 7 days.

Biochemical differences between the NML clinical isolates and those reported for the type species of the closely related genera of the family *Aerococcaceae* are summarized in Table 4. NML isolates were tested using API ZYM, API Strep 20 and API 50 CH kits as described by the manufacturer (https://www.biomerieux.com/us/en/our-offer/clinical-products/api.html) with all biochemical data for the NML strains in Table S5. Notably, the NML strains were negative for lipase (C14), *N*-acetyl-*β*-glucosaminidase, naphthol-AS-BI-phosphohydrolase, trypsin, valine arylamidase, *α*-chymotrypsin, *α*-fucosidase, *α*-glucosidase, *α*-mannosidase, *β*-glucosidase, alkaline phosphatase, *β*-galactosidase, *β*-glucuronidase, *α*-galactosidase, catalase and oxidase (Table S5). They were negative for the Voges–Proskauer test and hippurate production and positive for pyrrolidonyl arylamidase. The production of leucine aminopeptidase and arginine dihydrolase was variable, but activity was detected for the type strain NML130460^T^. Strains had the ability to ferment some substrates in the API 50 CH test, notably d-galactose, d-glucose, d-fructose, d-mannose, starch and glycogen, among others (Table S5).

In summary, the data from the polyphasic taxonomic analysis, which includes 16S rRNA phylogenetic analysis and WGS phylogenomic analysis as well as proteomic, phenotypic and chemotaxonomic data, showed that the 11 clinical isolates, mainly from wounds, form a separate monophyletic taxonomic lineage within the *Aerococcaceae* family and that they represent a novel genus for which the name *Bernardella* gen. nov. is proposed with *B. vulneris* sp. nov. as a species and NML130460^T^ as the type strain.

## Description of *Bernardella* gen. nov.

*Bernardella* (Ber.nar.del’la. N.L. fem. n. *Bernardella*, named after Kathryn Ann Bernard, a Canadian microbiologist, in recognition of her numerous contributions to microbiology).

Cells are Gram-stain-positive, non-motile cocci in pairs or clusters. Catalase-negative and oxidase-negative and facultatively anaerobic. The main cell wall fatty acids are the C_16:0_ and C_18:0_ saturated acids and the unsaturated C_18:1_ *ω*9c. The main diagnostic diamino acid is predicted to be l-Lys, based on the analysis of the MurE protein sequence. The *in silico* genome analysis of the glycerophospholipid metabolism predicts the production of phosphatidyl-l-serine.

*Bernardella* gen. nov*.* belongs to the family *Aerococcaceae* within the order *Lactobacillales*. The type species, isolated from a deep wound, is *B. vulneris*. G+C content of DNA is 42.3 mol% and genome size 1.9 Mb.

## Description of *Bernardella vulneris* (vul’ne.ris. L. gen. n. *vulneris*, of a wound)

Cells are Gram-stain-positive cocci occurring mainly in pairs. Cells growing on Columbia blood agar produce 0.5 mm, grey, circular, opaque, non-haemolytic colonies with entire edges after 24 h at 37 °C with 5% CO_2_. Strains grow scantily in NB with 6.5% (w/v) NaCl after 7 days at 37 °C. Facultatively anaerobic and catalase- and oxidase-negative. Grows at 25, 37 and 42 °C.

*B. vulneris* strains are negative for lipase (C14), *N*-acetyl-*β*-glucosaminidase, naphthol-AS-Bi-phosphohydrolase, trypsin, valine arylamidase, *α*-fucosidase, *α*-glucosidase, *α*-mannosidase, *β*-glucosidase, alkaline phosphatase, *β*-galactosidase, *β*-glucuronidase and *α*-galactosidase. They are negative for the Voges–Proskauer test and hippurate production. Positive for pyrrolidonyl arylamidase activity. The production of leucine aminopeptidase and arginine dihydrolase is variable, but activity was detected for the type strain NML130460^T^. Strains have the ability to ferment d-galactose, d-glucose, d-fructose and d-mannose, starch, glycogen, gentiobiose, d-trehalose, d-maltose, d-cellobiose, salicin, aesculin ferric citrate, *N*-acetyl glucosamine and arbutin. Strains may or may not have leucine arylamidase, *α*-chymotrypsin and *α*-glucosidase activity. The main cell wall fatty acids are the C_16:0_ and C_18:0_ saturated acids and the unsaturated C_18:1_ *ω*9c. The main diagnostic diamino acid is predicted to be l-Lys, based on the analysis of the MurE protein sequence.

The type strain of *B. vulneris* gen. nov., sp. nov. is NML130460^T^ (=LMG 32739^T^=NCTC 14840^T^), which was isolated from a deep wrist wound. The DNA G+C content of the type strain is 42.3 mol% and the genome size is 1.9 Mb. Ten additional strains of the species were isolated mainly from wounds. Whole-genome sequences of *B. vulneris* strains are available with accession numbers JAMXJD01–JAMXJN01.

## Supplementary material

10.1099/ijsem.0.007180 Supplementary Material 1.

## Abbreviations

AAI: average amino acid identity
ANIb: average nucleotide identity using blastn
CBAB: Columbia blood agar base
CFA: cellular fatty acid
dDDH: digital DNA–DNA hybridization
GGDC: Genome-to-Genome Distance Calculator
MALDI-TOF MS: matrix-assisted laser desorption/ionization time-of-flight MS
MSP: main spectral profile
NB: nutrient broth
NML: National Microbiology Laboratory
PE: paired-end
WGS: whole-genome sequencing
