# Supplementary material for: Description of Bernardella vulneris gen. nov., sp. nov., assignable to the family Aerococcaceae, isolated from human clinical samples
Source: Int J Syst Evol Microbiol. 2026 Aug 11;76(8):007180. doi: 10.1099/ijsem.0.007180 (PMC13472200; doi:10.1099/ijsem.0.007180)
Supplement: Supplementary file 1 [file ijsem-76-07180-s001.pdf]

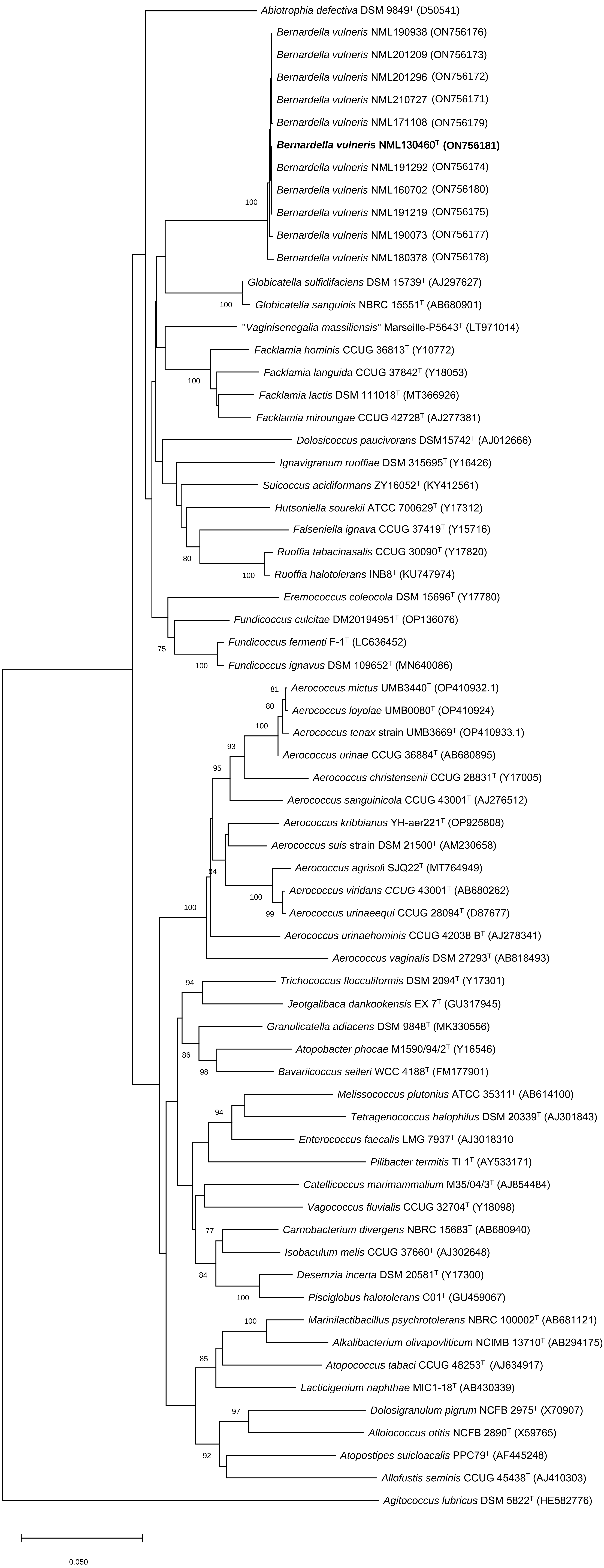

Supplementary Figure 1 - Phylogenetic consensus tree based on nearly full-length 16S rRNA gene sequences from the Aerococcaceae, Carnobacteriaceae and Enterococcaceae species aligned using Clustal W in MEGA X. The phylogeny was constructed with the neighbor-joining algorithm using 1000 resamplings with values ≥ 70% shown next to the branches. There were a total of 1575 positions in the final data set and the bar represents the number of substitutions per site.

Supplementary Figure 2 - Phylogenetic consensus tree based on nearly full-length 16S rRNA gene sequences from the Aerococacceae, Carnobacteriaceae and Enterococcaceae species aligned using Clustal W in MEGA X. The phylogeny was constructed with the Minimum Evolution method. The tree is drawn to scale and the percentage of replicate trees in which the associated taxa clustered together in the bootstrap test (1000 replicates) are shown next to the branches (≥70%). There were a total of 1575 positions in the final data set and the bar represents the number of substitutions per site.

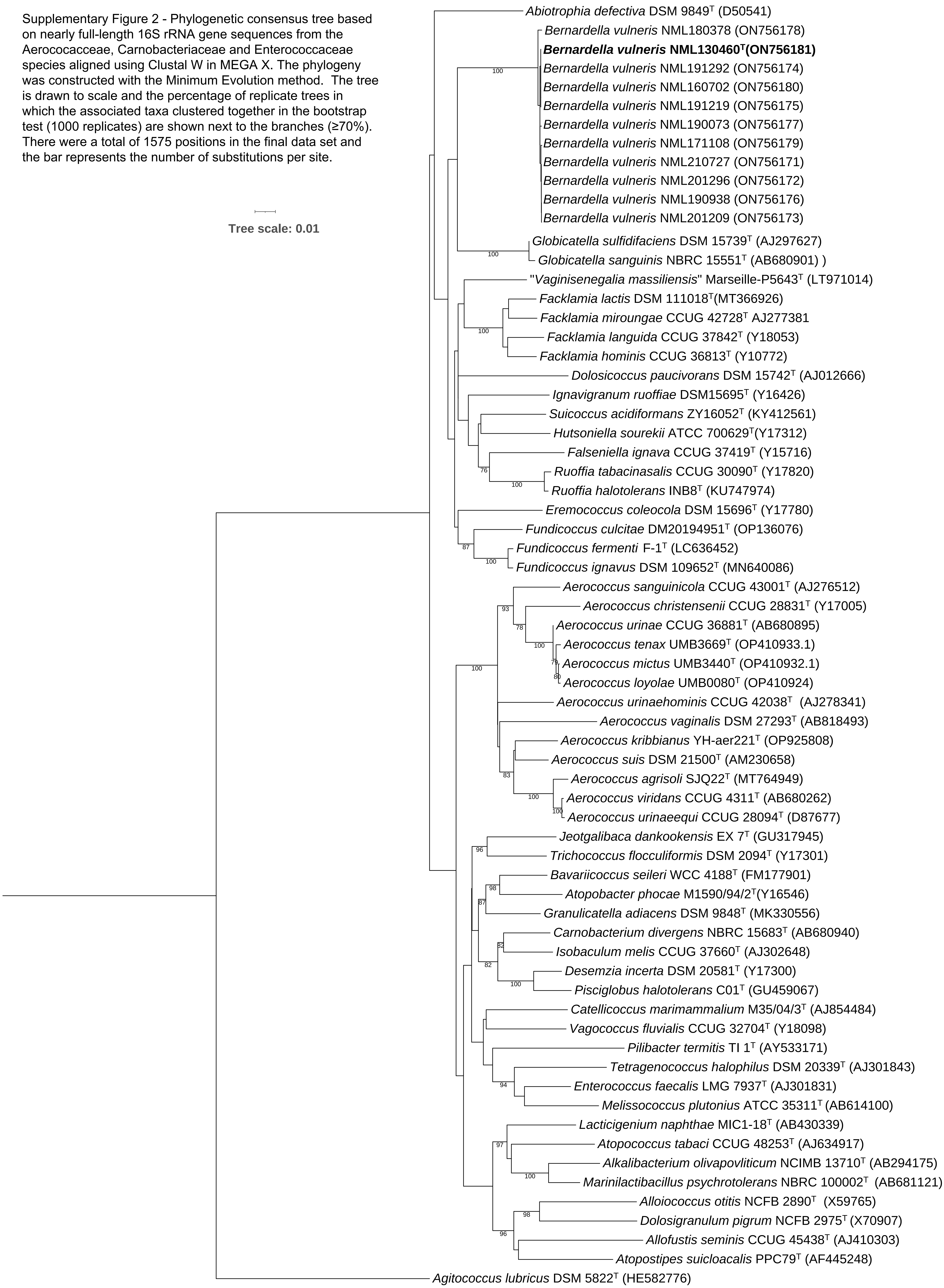

Supplementary Figure 3 - Glycerophospholipid metabolism pathways of Bernardella vulneris NML130460T as determined by annotation through KEGG Blast Koala (https://www.kegg.jp/blastkoala/)

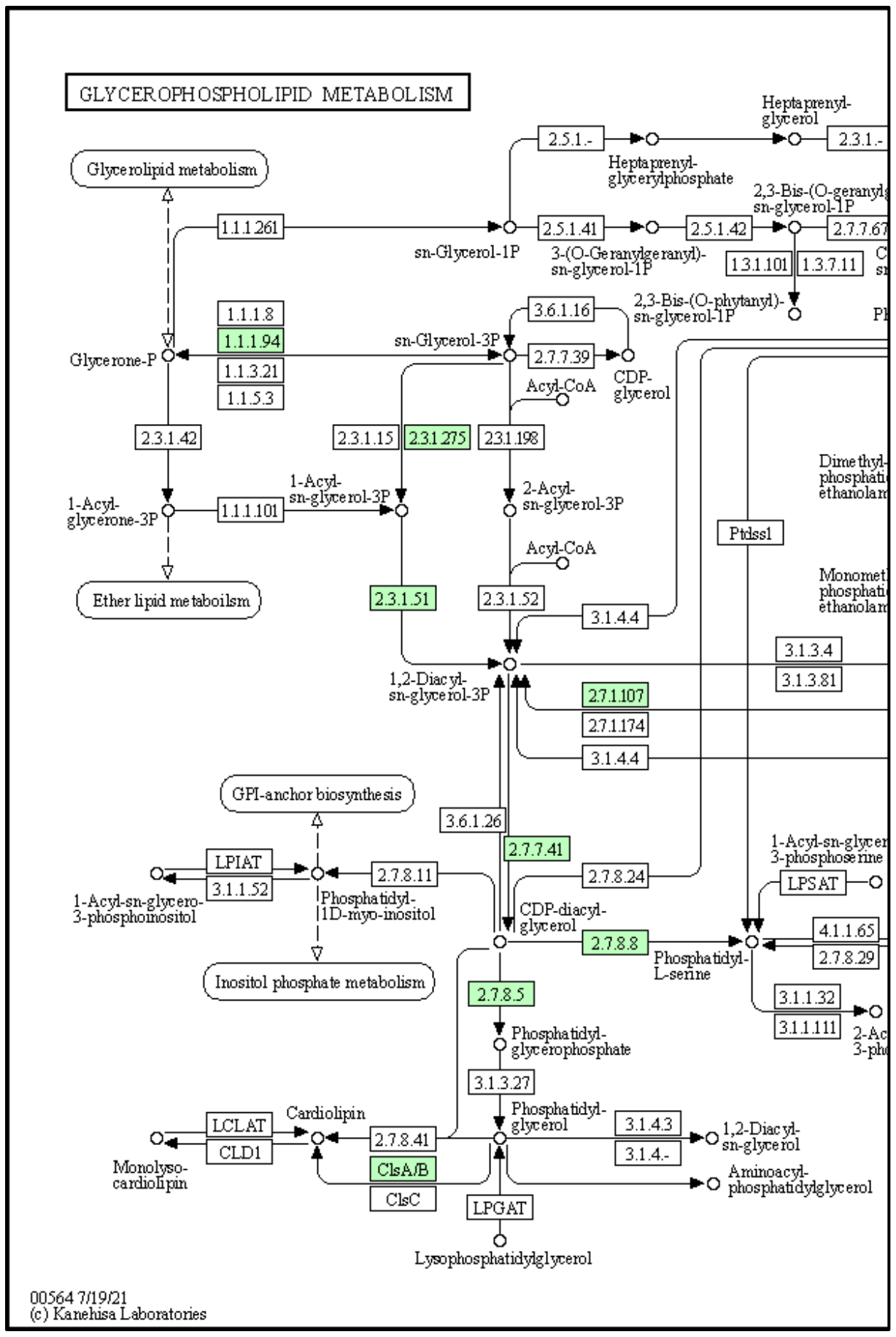

Supplemental figure 3 – Glycerophospholipid metabolism pathways of NML Bernardella vulneris 130460[T] as determined by annotation through KEGG Blast Koala (https://www.kegg.jp/blastkoala/)

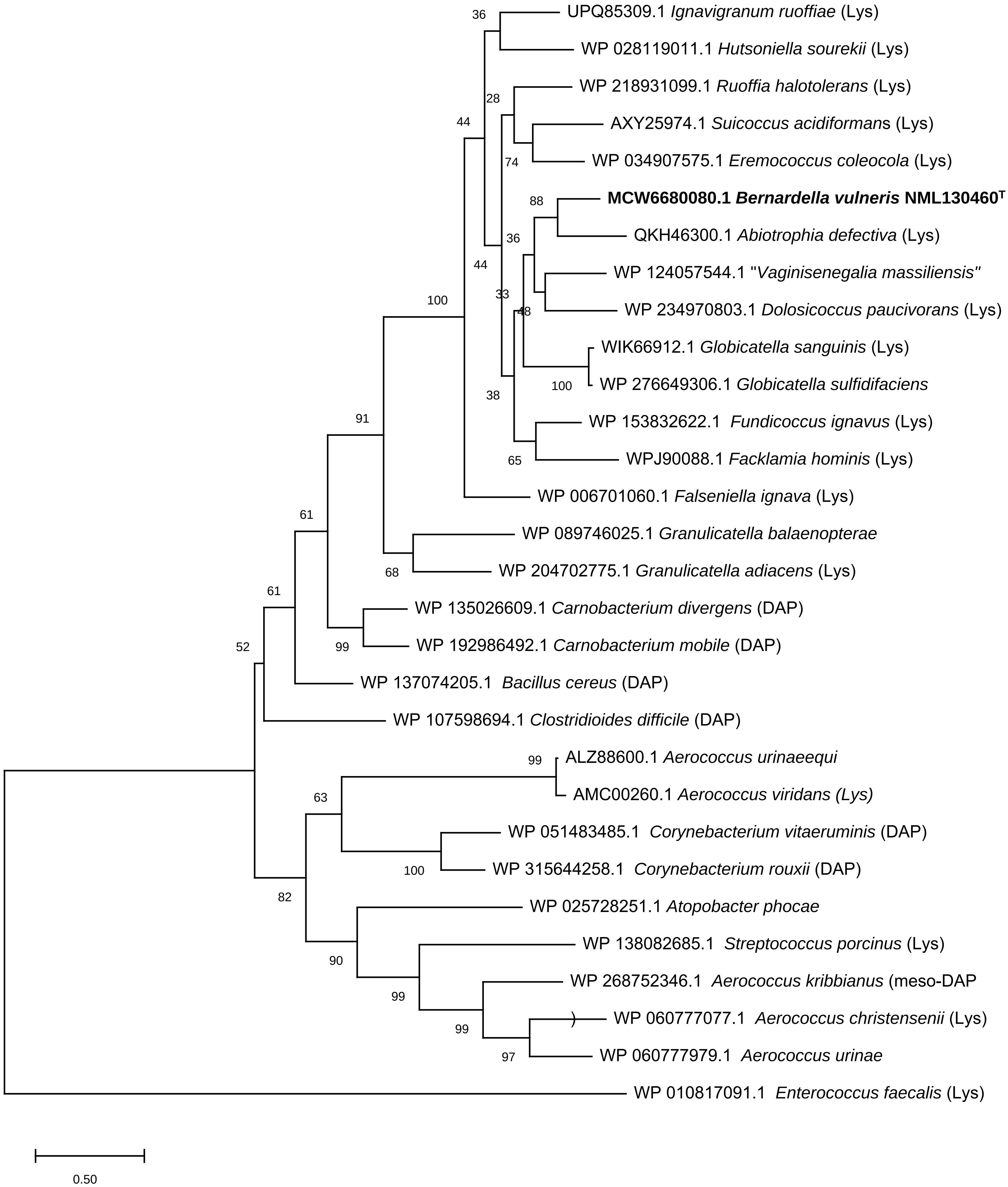

Supplementary Figure 4 - The evolutionary history was inferred by using the Maximum Likelihood method and JTT matrix-based model from the aligned protein sequences of MurE protein sequences from species related to Bernardella vulneris. The percentage of trees in which the associated taxa clustered together is shown next to the branches. The tree is drawn to scale, with branch lengths measured in the number of substitutions per site. There were a total of 554 positions in the final dataset. Evolutionary analyses were conducted in MEGA X. The main diamino acid for a species, when known, is in brackets.

Supplemental Table 1A) 16S rRNA gene BLASTN comparisons of all *Bernardella vulneris* isolates to each other. 1B) Overall genome relatedness analyses and 16S gene comparisons of the clinical isolates to the *Bernardella vulneris* type strain NML 130460$^{T.}$ 1C) Overall genome relatedness analyses and 16S gene Blast % ID comparisons of all *Aerococcaceae* species to *Bernardella vulneris* NML 130460^T (genome for *Aerococcus vaginalis* and *Fundicoccus fermenti* are unavailable).

A) 16S rRNA BLASTN results, all NML *Bernardella vulneris* isolates to each other.

	NML130460^T	NML160702	NML171108	NML180378	NML190073	NML190938	NML191219	NML191292	NML201209	NML201296	NML210727
NML130460^T	100.00	100.00	99.86	99.66	99.79	99.93	100.00	100.00	99.86	99.93	99.93
NML160702	100.00	100.00	99.86	99.66	99.79	99.93	100.00	100.00	99.86	99.93	99.93
NML171108	99.86	99.86	100.00	99.52	99.79	99.93	99.86	99.86	99.86	99.93	99.93
NML180378	99.66	99.66	99.52	100.00	99.45	99.59	99.66	99.66	99.52	99.59	99.59
NML190073	99.79	99.79	99.79	99.45	100.00	99.86	99.79	99.79	99.79	99.86	99.86
NML190938	99.93	99.93	99.93	99.59	99.86	100.00	99.93	99.93	99.93	100.00	100.00
NML191219	100.00	100.00	99.86	99.66	99.79	99.93	100.00	100.00	99.86	99.93	99.93
NML191292	100.00	100.00	99.86	99.66	99.79	99.93	100.00	100.00	99.86	99.93	99.93
NML201209	99.86	99.86	99.86	99.52	99.79	99.93	99.86	99.86	100.00	99.93	99.93
NML201296	99.93	99.93	99.93	99.59	99.86	100.00	99.93	99.93	99.93	100.00	100.00
NML210727	99.93	99.93	99.93	99.59	99.86	100.00	99.93	99.93	99.93	100.00	100.00

B) NML *B. vulneris* isolates compared to *B. vulneris* NML130460^T

***Bernardella vulneris* strain**	**Anib**	**dDDH**	**AAI**	**16S**
NML160702	97.71	81.00	97.42	100.00
NML171108	98.16	84.10	98.66	99.86
NML180378	97.14	76.30	97.74	99.66
NML190073	97.29	77.30	96.87	99.79
NML190938	97.44	78.00	97.51	99.93
NML191219	97.01	75.30	97.06	100.00
NML191292	97.68	81.80	98.17	100.00
NML201209	97.41	77.70	97.83	99.86
NML201296	97.41	78.20	97.61	99.93
NML210727	97.41	78.20	97.57	99.93

16S %ID by NCBI BLASTN
ANIb values calculated by JSpeciesWS https://jspecies.ribohost.com/jspeciesws/
dDDH values for formula 2 calculated by Genome to Genome distance calculator https://ggdc.dsmz.c
AAI values calculated by Average amino acide identity calculator http://enve-omics.ce.gatech.edu/a

**C) BLASTN % ID to all type species of family *Aerococcaceae* to NML130460^T**				
**Species**	**ANIb**	**dDDH**	**AAI**	**16S**
Abiotrophia defectiva DSM 9849^T	68.64	27.30	62.13	92.81
Aerococcus agrisoli SJQ22^T	65.50	17.40	48.61	88.46
Aerococcus christensenii CCUG 28831^T	65.21	28.50	47.84	89.12
Aerococcus kribbianus YH-aer221^T	65.21	28.50	49.24	89.95
Aerococcus loyolae UMB0080^T	64.55	29.40	47.71	90.04
Aerococcus mictus UMB3440^T	64.34	29.40	47.51	90.07
Aerococcus sanguinicola CCUG 43001^T	64.73	28.60	49.04	89.80
Aerococcus suis DSM 21500^T	65.60	19.50	49.67	90.31
Aerococcus tenax UMB3669^T	64.57	27.60	47.71	90.12
Aerococcus urinae CCUG 36881^T	64.36	29.40	47.73	90.35
Aerococcus urinaeequi CCUG 28094^T	65.68	21.70	49.04	88.99
Aerococcus urinaehomini s CCUG 42038^T	65.15	26.30	49.41	90.78
Aerococcus vaginalis DSM 27293^T	N/A	N/A	N/A	89.08
Aerococcus viridans CCUG 4311^T	65.65	22.00	48.91	88.68
Dolosicoccus paucivorans DSM 15742^T	66.86	22.60	55.81	91.84
Eremococcus coleocola DSM 15696^T	66.64	23.80	56.45	91.40
Facklamia hominis CCUG 36813^T	65.70	24.00	53.77	91.71
Facklamia lactis DSM 111018^T	66.09	22.20	55.00	92.94
Facklamia languida CCUG 37842^T	65.91	22.70	55.47	92.27
Facklamia miroungae CCUG 42728^T	66.27	21.90	54.76	92.05
Falseniella ignava CCUG 37419^T	66.46	20.70	54.19	90.47
Fundicoccus culcitae DM20194951^T	67.65	23.40	57.15	92.95
Fundicoccus fermenti F-1^T	N/A	N/A	N/A	93.30
Fundicoccus ignavus DSM 109652^T	68.71	21.10	59.85	92.74
Globicatella sanguinis NBRC 15551^T	68.69	23.00	59.38	93.34
Globicatella sulfidifacien s DSM 15739^T	68.75	20.10	59.08	92.79
Hutsoniella sourekii ATCC 700629^T	65.94	19.70	55.10	91.38
Ignavigranum ruoffiae DSM 15695^T	66.63	23.40	55.01	91.20
Ruoffia halotolerans INB8^T	67.27	23.30	57.00	91.04
Ruoffia tabacinasalis CCUG 30090^T	67.31	23.20	56.89	91.31
Suicoccus acidiformans ZY16052^T	66.45	26.10	55.41	91.35
'*Vaginisenegalia massiliensis* '' Marseille-F	68.92	27.40	60.40	92.78

16S %ID by NCBI BLASTN
ANIb values calculated by JSpeciesWS https://jspecies.ribohost.com/jspeciesws/
dDDH values for formula 2 calculated by Genome to Genome distance calculator https://ggdc.dsmz.de/distcalc2.php
AAI values calculated by Average amino acide identity calculator http://enve-omics.ce.gatech.edu/aai/

Supplementary Table 2 - Genome assembly and annotation statistics for all *Bernardella vulneris* isolates

Isolate	Coverage (x)	Base pairs	G+C content (mol%)	Contigs	Contig N50 (kb)	CDS	rRNA	tRNA	repeats
NML130460[T]	107	1,903,664	42.33%	25	224.3	1711	5	43	4
NML160702	125	2,069,421	41.97%	29	126.1	1955	4	38	5
NML171108	112	1,888,377	42.10%	65	70.8	1677	5	33	4
NML180378	126	1,890,407	42.07%	40	96.3	1685	5	42	5
NML190073	194	1,957,334	42.42%	19	182.3	1789	4	38	1
NML190938	169	1,885,894	42.19%	31	137.1	1694	5	43	3
NML191219	156	2,041,755	41.80%	29	135.6	1879	5	43	4
NML191292	239	1,973,186	41.99%	46	196.6	1745	7	42	3
NML201209	110	1,858,797	42.39%	37	79.2	1660	4	38	5
NML201296	144	1,872,056	42.21%	21	195.6	1667	5	41	3
NML210727	117	1,878,929	42.21%	65	56.1	1683	4	37	3

Supplementary Table 3 - CRISPR arrays found in all *Bernardella vulneris* strains via https://crisprcas.i2bc.paris-saclay.fr/. Identical consensus repeat are in the same colour.

Strain	Sequence	Duplicated Spacers	CRISPR Start	CRISPR End	CRISPR Length	Potential Orientation (AT%)	Consensus Repeat	Repeat ID (CRISPRdb)	Nb CRISPRs with same Repeat (CRISPRdb)	Repeat Length	Spacers Nb	Mean size Spacers	Standard Deviation Spacers	# Repeats matching Consensus	Ratio Repeats match/TotalRepeat	Conservation Repeats (% identity)	Ebcons Repeats*	Conservation Spacers (% identity)	EBcons Spacers	Repeat Length plus mean size Spacers	Ratio Repeat/mean Spacers Length	CRISPRfound in DB (if sequence IDs are similar)	Evidence Level
NML130460[T]	Contig00001 len 455235 cov 42	1	165683	169810	4127	Forward	GTTGGACACTCATTCAAAATAACATGACTCTAAAAC	Unknown	0	36	62	30.0	0.0	63.0	1.0	100.0	100.0	0.0	0.0	66.0	1.2	0	4
NML130460	Contig00001 len 455235 cov 42	0	273458	274726	1268	Reverse	GTTTCAATCCCCTCACGGAGGTGATGGGTTTCTTAT	Unknown	0	36	17	36.5	1.7	17.0	0.9	97.2	99.1	0.0	0.0	72.5	1.0	0	4
NML130460	Contig00002 len 371563 cov 45	1	977	3817	2840	Reverse	GTTTCAATCCCCTCACGGGGTGGAGGGGGTTGCTAT	Unknown	0	36	39	35.9	1.8	31.0	0.8	55.3	85.2	0.0	0.0	71.9	1.0	0	4
NML130460	Contig00004 len 211587 cov 41	0	6666	7768	1102	Forward	GTTTCAATCCCCTCACGGGGTGGAGGGGGTTGCTAT	Unknown	0	36	15	35.1	1.8	11.0	0.7	65.0	87.3	0.0	0.0	71.1	1.0	0	4
NML160702	Contig00001 len 262365 cov 41	0	72327	72889	562	Unknown	GTTTTAGAGTCATGTTATTTTGAATGAGTGTCCAAC	Unknown	0	36	8	29.9	0.4	7.0	0.8	52.8	76.2	0.0	0.0	65.9	1.2	0	4
NML160702	Contig00001 len 262365 cov 41	0	93328	93759	431	Reverse	GTTTTAGAGTCATGTTATTTTGAATGAGTGTCCAAC	Unknown	0	36	6	30.0	0.0	7.0	1.0	100.0	100.0	2.6	0.0	66.0	1.2	0	4
NML160702	Contig00002 len 242344 cov 50	1	93996	105739	11743	Forward	GTTTTAGAGTCATGTTATTTTGAATGAGTGTCCAAC	Unknown	0	28	192	33.0	0.1	170.0	0.9	67.9	96.7	0.0	78.0	61.0	0.8	0	4
NML160702	Contig00017 len 30948 cov 49	0	17451	18921	1470	Forward	ATAAGAAACCCGTCACCTCCGTGAGGGGATTGAAAC	Unknown	0	36	20	35.8	1.5	8.0	0.4	80.6	83.4	0.0	0.0	71.8	1.0	0	4
NML160702	Contig00021 len 12601 cov 35	0	5797	6271	474	Reverse	GTTTCAATCCCCTCGCGGGGCGGTGGGGGTTTCTATT	Unknown	0	37	6	36.0	2.2	2.0	0.3	51.2	72.0	6.3	0.0	73.0	1.0	0	4
NML171108	Contig00005 len 82344 cov 37	0	45947	47369	1422	Forward	GTTGGACACTCATTCAAAATAACATGACTCTAAAAC	Unknown	0	36	21	30.0	0.2	22.0	1.0	100.0	100.0	0.0	0.0	66.0	1.2	0	4
NML171108	Contig00007 len 81046 cov 48	0	61475	63664	2189	Reverse	GTTTCAATCCCCTCACGGAGGTGATGGGTTTCTTAT	Unknown	0	36	30	35.8	1.5	28.0	0.9	97.2	98.7	0.0	82.0	71.8	1.0	0	4
NML171108	Contig00010 len 70784 cov 50	0	21398	22216	818	Forward	ATAGCAACCCCCTCCACCCCGTGAGGGGATTGAAACC	Unknown	0	37	11	34.1	2.1	4.0	0.3	67.6	78.8	0.0	0.0	71.1	1.1	0	4
NML171108	Contig00035 len 10783 cov 27	0	2960	5881	2921	Reverse	GTTTCAATCCCCTCGCGGGGCGGAGGGGTTTTCTAT	Unknown	0	36	40	36.2	1.8	40.0	1.0	79.5	97.9	0.0	0.0	72.2	1.0	0	4
NML180378	Contig00001 len 224445 cov 41	0	201442	202028	586	Forward	CCACCTCGTGAGGGGATTGAAAC	Unknown	0	23	8	47.5	1.8	5.0	0.6	91.3	92.7	16.9	1.6	70.5	0.5	0	3
NML180378	Contig00006 len 102676 cov 32	0	31129	31492	363	Unknown	GTTTTAGAGTCATGTTATTTTGAATGAGTGTCCAAC	Unknown	0	36	5	29.6	0.9	4.0	0.7	58.3	72.9	5.1	0.1	65.6	1.2	0	4
NML180378	Contig00006 len 102676 cov 32	0	51930	55660	3730	Reverse	GTTTTAGAGTCATGTTATTTTGAATGAGTGTCCAAC	Unknown	0	36	56	30.0	0.1	57.0	1.0	100.0	100.0	0.0	0.0	66.0	1.2	0	4
NML180378	Contig00009 len 80535 cov 44 1	1	17417	18745	1328	Forward	ATAAGAAACCCATCACCTCCGTGAGGGGATTGAAAC	Unknown	0	36	18	35.8	1.7	17.0	0.9	94.4	98.3	0.0	0.0	71.8	1.0	0	4
NML180378	Contig00028 len 12609 cov 28	0	5388	6223	835	Reverse	GTTTCAATCCCCTCGCGGGGCGGAGGGGTTTTCTAT	Unknown	0	36	11	36.7	1.8	11.0	0.9	79.5	94.7	0.0	0.0	72.7	1.0	0	4
NML190073	Contig00002 len 202057 cov 39	0	144201	145359	1158	Reverse	GTTTTAGAGTCATGTTATTTTGAATGAGTGTCCAAC	Unknown	0	36	17	30.1	0.2	17.0	0.9	69.4	90.5	0.0	0.0	66.1	1.2	0	4
NML190938	Contig00002 len 185118 cov 39	0	17415	19597	2182	Forward	ATAAGAAACCCATCACCTCCGTGAGGGGATTGAAAC	Unknown	0	36	30	35.6	1.5	30.0	1.0	97.2	99.4	0.0	0.0	71.6	1.0	0	4
NML190938	Contig00002 len 185118 cov 39	0	126645	128462	1817	Reverse	GTTTTAGAGTCATGTTATTTTGAATGAGTGTCCAAC	Unknown	0	36	27	30.0	0.0	27.0	1.0	69.4	93.2	0.0	0.0	66.0	1.2	0	4
NML190938	Contig00006 len 137100 cov 34	0	129810	130125	315	Reverse	GTTTCAATCCCCTCGCGGGGCGGAGGGG	Unknown	0	28	4	44.0	2.0	5.0	1.0	100.0	100.0	15.8	23.6	72.0	0.6	0	3
NML191219	Contig00001 len 314885 cov 24	0	152437	152935	498	Forward	TGTTGGACACTCATTCAAAATAACATGACTCTAAAAC	Unknown	0	37	7	29.0	0.0	6.0	0.8	97.3	97.1	0.0	0.0	66.0	1.3	0	4
NML191219	Contig00001 len 314885 cov 24	1	312954	314866	1912	Unknown	GTTTCAATCCCCTCACGGAGGTGATGGGTTTCTTAT	Unknown	0	36	26	36.2	1.4	26.0	1.0	97.2	99.4	0.0	0.0	72.2	1.0	0	4
NML191219	Contig00004 len 151142 cov 28	0	22	698	676	Unknown	GTTTCAATCCCCTCACGGAGGTGATGGGTTTCTTAT	Unknown	0	36	9	35.2	0.7	10.0	1.0	100.0	100.0	0.0	0.0	71.2	1.0	0	4
NML191219	Contig00004 len 151142 cov 28	0	135375	136440	1065	Unknown	GGATCACCCCCGCGTATGCGGGTAGCAG	Unknown	0	28	17	33.1	0.2	16.0	0.9	78.6	89.5	0.0	0.0	61.1	0.8	0	4
NML191292	Contig00002 len 363150 cov 30	1	186668	188842	2174	Forward	ATAAGAAACCCATCACCTCCGTGAGGGGATTGAAAC	Unknown	0	36	30	35.3	1.5	31.0	1.0	100.0	100.0	0.0	0.0	71.3	1.0	0	4
NML191292	Contig00002 len 363150 cov 30	1	292394	295321	2927	Reverse	GTTTTAGAGTCATGTTATTTTGAATGAGTGTCCAAC	Unknown	0	36	43	31.3	8.4	43.0	1.0	91.7	98.7	0.0	0.0	67.3	1.2	0	4
NML191292	Contig00005 len 165219 cov 28	0	131884	132266	382	Unknown	GTTTCAATCCCCTCGCGGGGCGGAGGGGTTTTCTAT	Unknown	0	36	5	33.4	2.2	4.0	0.7	67.6	80.7	4.2	7.0	69.4	1.1	0	4
NML201209	Contig00001 len 257253 cov 38	1	211389	212047	658	Reverse	ATAAGAAACCCATCACCTCCGTGAGGGGATTGAAAC	Unknown	0	28	9	42.1	1.5	4.0	0.4	64.3	78.5	13.0	0.0	70.1	0.7	0	3
NML201209	Contig00002 len 182492 cov 38	1	14704	17409	2705	Unknown	CTGCTACCCGCATACGCGGGGGTGATCC	Unknown	0	28	44	32.9	1.9	37.0	0.8	78.6	92.6	0.0	0.0	60.9	0.9	0	4
NML201209	Contig00003 len 182159 cov 34	1	150206	150703	497	Forward	GTTGGACACTCATTCAAAATAACATGACTCTAAAAC	Unknown	0	36	7	30.0	0.0	7.0	0.9	97.2	98.5	2.9	0.0	66.0	1.2	0	4
NML201209	Contig00015 len 49518 cov 42	0	17377	18773	1396	Forward	ATAAGAAACCCATCACCTCCGTGAGGGGATTGAAAC	Unknown	0	36	19	35.6	1.5	19.0	1.0	97.2	99.2	0.0	0.0	71.6	1.0	0	4
NML201209	Contig00016 len 35589 cov 31	0	7475	8229	754	Reverse	GTTTCAATCCCCTCGCGGGGCGGAGGGGTTTTCTAT	Unknown	0	36	10	35.9	1.2	10.0	0.9	75.0	89.0	0.0	0.0	71.9	1.0	0	4
NML201296	Contig00001 len 248573 cov 24	0	231732	233899	2167	Forward	GGATCACCCCCGCGTATGCGGGTAGCAG	Unknown	0	28	35	33.1	0.4	34.0	0.9	78.6	95.6	0.0	0.0	61.1	0.8	0	4
NML201296	Contig00003 len 208053 cov 21	0	173088	173518	430	Forward	GTTGGACACTCATTCAAAATAACATGACTCTAAAAC	Unknown	0	36	6	29.8	0.4	6.0	0.9	72.2	83.6	5.3	0.0	65.8	1.2	0	4
NML201296	Contig00003 len 208053 cov 21	0	184476	187284	2808	Forward	GTTGGACACTCATTCAAAATAACATGACTCTAAAAC	Unknown	0	36	42	30.0	0.2	42.0	1.0	69.4	95.1	0.0	0.0	66.0	1.2	0	4
NML210727	Contig00005 len 75877 cov 42	0	14675	16841	2166	Reverse	CTGCTACCCGCATACGCGGGGGTGATCC	Unknown	0	28	35	33.1	0.3	34.0	0.9	78.6	95.6	0.0	0.0	61.1	0.8	0	4
NML210727	Contig00010 len 68598 cov 35	0	6144	10206	4062	Reverse	GTTTTAGAGTCATGTTATTTTGAATGAGTGTCCAAC	Unknown	0	36	61	30.0	0.1	61.0	1.0	69.4	96.4	0.0	0.0	66.0	1.2	0	4
NML210727	Contig00010 len 68598 cov 35	0	21164	21594	430	Reverse	GTTTTAGAGTCATGTTATTTTGAATGAGTGTCCAAC	Unknown	0	36	6	29.8	0.4	6.0	0.9	72.2	83.6	5.1	0.0	65.8	1.2	0	4

*EBcons are a measure of CRISPR repeat conservation. Putative CRISPR arrays with at least four spacers are assigned to levels 2–4 as follows: repeats EBcons < 70 (level 2), repeats EBcons ≥ 70 and spacers overall percentage identity > 8% (level 3); repeats EBcons ≥ 70 and spacers overall percentage identity ≤ 8% (level 4). CRISPR arrays having evidence-levels 3 and 4 may be considered as highly likely candidates, whereas evidence-levels 1 and 2 indicate potentially invalid CRISPR arrays.

Supplementary Table 4 - Minimum inhibitory concentrations (MICs) for *Bernardella vulneris* NML130460[T] from antimicrobial susceptibility assessed using the broth microdilution method on Sensititre GPN3F and STP6F plates

**Class**	**antimicrobial agent**	**MIC**
Penicillins	Penicillin	≤ 0.03
Cephems	Cefotaxime	≤ 0.5
Cephems	Ceftriaxone	≤ 2.0
Carbapenems	Meropenem	≤ 0.25
Glycopeptides	Vancomycin	≤ 1
Fluoroquinolones	Ciprofloxacin	≤ 0.5
Fluoroquinolones	Levofloxacin	≤ 0.25
Tetracyclines	Tetracycline	≤ 2
Folate pathway inhib	Trimethoprim-sulfamethoxaz	≤ 0.5/9.5
Oxazolidinones	Linezolid	≤ 2
**Others**		
Lincosamides	Clindamycin	≤ 2
Cephems	Cefuroxime	≤ 2
Fluoroquinolones	Moxifloxacin	≤ 1
Macrolides	Azithromycin	≤ 0.25
Carbapenems	Ertapenem	≤0.5
Macrolides	Erythromycin	≤ 0.25
Penicillins	Amoxicillin/clavulanic acid	≤ 2/1
Lipopeptides	Daptomycin	≤ 0.25
Cephalosporins	Cefepime	≤ 2
Phenicols	Chloramphenicol	≤ 4
Tetracyclines	Tigecycline	≤ 0.015
Aminoglycosides	Gentimicin	≤ 4
Streptogramins	Quinupristin/Dalfopristin	≤ 0.25
Penicillins	Ampicillin	≤ 0.12
Ansamycins	Rifampin	≤ 0.5
Fluoroquinolones	Gatifloxacin	≤ 1
Penicillins	Oxacillin	≤ 0.25

Supplementary Table 5 - Biochemical analysis of all *Bernardella vulneris* isolates from APIZYM, API CH50 and API Strep 20 kits from Biomérieux.

API 50 CH	Substrate/Enzyme/Reaction	NML130460^{T}	NML160702	NML180378	NML190073	NML210727	NML171108	NML190938	NML191219	NML191292	NML201209	NML201296	*Summary n=11	Final interpretation
	Glycerol	-	-	-	-	-	-	-	-	-	-	-	-	-
	Erythritol	-	-	-	-	-	-	-	-	-	-	-	-	-
	D-arabinose	-	-	-	-	-	-	-	-	-	-	-	-	-
	D-xylose	-	-	-	-	-	-	-	-	-	-	-	-	-
	L-xylose	-	-	-	-	-	-	-	-	-	-	-	-	-
	D-adonitol	-	-	-	-	-	-	-	-	-	-	-	-	-
	Methyl-ßD-xylopyranoside	-	-	-	-	-	-	-	-	-	-	-	-	-
	D-galactose	+	+	+	+	+	+	+	+	+	+	+	+	+
	D-glucose	+	+	+	+	+	+	+	+	+	+	+	+	+
	D-fructose	+	+	+	+	+	+	+	+	+	+	+	+	+
	D-mannose	+	+	+	+	+	+	+	+	+	+	+	+	+
	L-sorbose	-	-	-	-	-	-	-	-	-	-	-	-	-
	L-rhamnose	-	-	-	-	-	-	-	-	-	-	-	-	-
	Dulcitol	-	-	-	-	-	-	-	-	-	-	-	-	-
	Inositol	-	-	-	-	-	-	-	-	-	-	-	-	-
	Methyl-αD-mannopyranoside	-	-	-	-	-	-	-	-	-	-	-	-	-
	Methyl-αD-glucopyranoside	-	-	-	-	-	-	-	-	-	-	-	-	-
	N-acetylglucosamine	+	+	+	+	+	+	+	+	+	+	+	+	+
	Amygdalin	+	+	+	+	+	-	+	+	+	+	+	10+/1-	v
	Arbutin	+	+	+	+	+	-	-	-	+	+	w. +	8+/3-	v
	Salicin	+	+	+	+	+	+	+	+	+	+	+	+	+
	D-cellobiose	+	+	+	+	+	+	+	+	+	+	+	+	+
	D-maltose	+	+	+	+	+	+	+	+	+	+	+	+	+
	D-melibiose	-	-	-	-	-	-	-	-	-	-	-	-	-
	D-saccharose (sucrose)	-	-	-	-	-	-	-	-	+	-	-	1+/10-	v
	D-melezitose	-	-	-	-	-	-	-	-	-	-	-	-	-
	Xylitol	-	-	-	-	-	-	-	-	-	-	-	-	-
	Gentiobiose	+	+	+	+	+	+	+	+	+	+	+	+	+
	D-Turanose	-	-	-	-	-	-	-	-	-	-	-	-	-
	D-lyxose	-	-	-	-	-	-	-	-	-	-	-	-	-
	D-tagatose	-	-	-	-	-	-	-	-	-	-	-	-	-
	D-fucose	-	-	-	-	-	-	-	-	-	-	-	-	-
	L-fucose	-	-	-	-	-	-	-	-	-	-	w. +	1w+/10-	v
	D-arabitol	-	-	-	-	-	-	-	-	-	-	-	-	-
	L-arabitol	-	-	-	-	-	-	-	-	-	-	-	-	-
	Potassium gluconate	-	-	-	-	-	-	-	-	-	-	-	-	-
	Potassium 2-ketogluconate	-	-	-	-	-	-	-	-	-	-	-	-	-
API 50 CH AND STREPT 20														
	Potassium 5-ketogluconate	w +	+	+	+	-	+	+	+	+	-	+	9+/2-	v
	Esculin ferric citrate	+/+	+/+	+/+	+/+	+/+	+/+	+/+	+/+	+/+	+/+	+/+	+	+
	L-arabinose	-/-	-/-	-/-	-/-	-/-	-/-	-/-	-/-	-/-	-/-	-/-	-	-
	D-ribose	-/-	-/-	-/-	-/-	-/-	-/-	-/-	-/-	-/-	-/-	-/-	-	-
	D-mannitol	-/-	-/-	-/-	-/-	-/-	-/-	-/-	-/-	-/+	-/-	-/-	-	-
	D-sorbitol	-/-	-/-	-/-	-/-	-/-	-/-	-/-	-/-	-/-	-/-	-/-	-	-
	D-lactose (bovine origin)	+/+	+/-	+/+	+/+	+/+	+/+	+/+	+/+	+/+	+/+	+/+	+	
	D-trehalose	+/-	+/-	+/-	+/-	+/+	+/+	+/+	+/+	+/+	+/+	w. +/+	7+/4+-	v
	Inulin	-/-	-/-	-/-	-/-	-/-	-/-	-/-	-/-	+/+	-/-	-/-	1+/10-	v
	D-raffinose	-/-	-/-	-/-	-/-	-/-	-/-	-/-	-/-	+/+	-/-	-/-	1+/10-	v
	Amidon (starch)	+/-	+/-	+/-	+/-	+/+	-/+	+/+	+/+	+/+	+/+	+/+	6+/5+-	v
	Glycogen	+/+	+/-	+/-	+/-	+/+	+/+	+/+	+/+	+/+	+/+	+/+	8+/3+-	v
API STREP 20														
	Acetoin production (Voges Proskauer)	-	-	-	-	-	-	-	-	-	-	-	-	-
	Hippuric acid hydrolysis	-	-	-	-	-	-	-	-	-	-	-	-	-
	Pyrrolidonyl arylamidase	+	+	+	+	+	+	+	+	+	+	+	+	+
	Leucine aminopeptidase	+	-	-	-	-	+	+	-	+	+	+	6+/5-	v
	Arginine dihydrolase	+	-	-	-	-	+	-	-	-	+	-	3+/8-	v
API STREP 20 AND APY ZYM														
	Alkaline phosphatase	-/-	-/-	-/-	-/-	-/-	-/-	-/-	-/-	-/-	-/-	-/-	-	-
	ß-galactosidase	-/-	-/-	-/-	-/-	-/-	-/-	-/-	-/-	-/-	-/-	-/-	-	-
	ß-glucuronidase	-/-	-/-	-/-	-/-	-/-	-/-	-/-	-/-	-/-	-/-	-/-	-	-
API Zym														
	α-galactosidase	-/-	-/-	-/-	-/-	-/-	-/-	-/-	-/-	-/-	-/-	-/-	-	-
	Acid phosphatase	-	-	-	-	-	-	-	-	-	-	-	-	-
	Cystine arylamidase	-	-	-	-	-	-	-	-	-	-	-	-	-
	Esterase (C 4)	-	-	-	-	-	-	-	-	-	-	-	-	-
	Esterase Lipase (C 8)	-	-	-	-	-	-	-	-	-	-	-	-	-
	Leucine arylamidase	+	+	+	-	+	-	-	-	+	-	-	5+/6-	v
	Lipase (C 14)	-	-	-	-	-	-	-	-	-	-	-	-	-
	N-acetyl-β-glucosaminidase	-	-	-	-	-	-	-	-	-	-	-	-	-
	Naphthol-AS-BI-phosphohydrolase	-	-	+	-	-	-	-	-	-	-	-	1+/10-	v
	Trypsin	-	-	-	-	-	-	-	-	-	-	-	-	-
	Valine arylamidase	-	-	-	-	-	-	-	-	-	-	-	-	-
	α-chymotrypsin	-	+	-	-	-	-	-	-	+	-	-	9-/2+	v
	α-fucosidase	-	-	-	-	-	-	-	-	-	-	-	-	-
	α-glucosidase	-	-	-	-	-	-	-	-	+	-	-	10-/1+	v
	α-mannosidase	-	-	-	-	-	-	-	-	-	-	-	-	-
	β-glucosidase	-	-	-	-	-	-	-	-	-	-	-	-	-
	Catalase	-	-	-	-	-	-	-	-	-	-	-	-	-
	Oxidase	-	-	-	-	-	-	-	-	-	-	-	-	-

+/- differing results for different tests
w + = weak positive
* number of positive/negative/ or positive and differing results for different results (+-)
v = variable results as final interpretation
